# Consumption and income expectations during Covid-19

**DOI:** 10.1007/s11150-023-09656-8

**Published:** 2023-04-22

**Authors:** Giovanni Immordino, Tullio Jappelli, Tommaso Oliviero

**Affiliations:** Department of Economics and Statistics and CSEF, University of Naples Federico II, Napoli, NA Italy

**Keywords:** D14, D15, Consumption Expectations, Income Expectations, Covid-19 Crisis

## Abstract

Using a survey of Italian households administered in November 2021, we study the effect of microeconomic and macroeconomic expectations about the health crisis and income growth on consumption expectations in 2022. The survey elicits individual-level indicators of income and consumption expectations, distinguishing between consumption at home, away from home, online and total. We find that expected household income and expected GDP growth are strongly related to consumption expectations; income risk is positively associated with expected consumption growth for richer households. Finally, our results indicate that health-related variables were not a major drivers of consumption expectations in 2022.

## Introduction

Following the deep, double-digit recession in 2020, the euro area economies, including Italy, recovered in 2021-22. In its most recent Economic Bulletin, the Bank of Italy (2023) reports that after a strong, demand-driven growth in 2021, household consumption continued to grow in 2022, but at a slower pace. Although the decline in consumption in 2020 and the subsequent recovery were sharp, movements in disposable income have been milder due to the government support policies in place during the crisis. The propensity to save reflects these movements, rising sharply from an average of 8% in the pre-Covid-19 period (2012-19) to almost 17% in the second quarter of 2020, then declining to 11% in 2021, and returning to pre-pandemic levels in 2022 (7.1%).

During the crisis, questions arose about how income expectations, expectations about Covid-19 cases, and social distancing measures would affect consumption expectations. To investigate these issues, with the help of Doxa, a leading institute for opinion polls, market research and statistical analysis, we designed and administered an Income and Spending Expectations Covid-19 (ISEC) survey. The survey collected information on over 3,000 Italian households, based on a questionnaire that asked, in November 2021, about standard socioeconomic variables and expectations about consumption, income and health in 2022. Notice that our survey was taken after the peak of the pandemic, during the 2021 recovery, which also featured a massive vaccination campaign (by the end of 2021 about 80% of the target population was vaccinated).

Our goal is to relate consumption plans for 2022 not just to mean income and health outcomes but to their distribution, therefore including measures of perceived idiosyncratic risk. For this reason, ISEC asked for respondents’ subjective expectations and probabilities of the distribution of consumption and income growth one year ahead, distinguishing between disposable income and aggregate GDP growth. We also asked for respondents’ expectations about the probability of future lockdowns, vaccination rates and fear of infection from Covid-19. The survey also included questions related to standard socioeconomic variables such as respondent age, family size and composition, education, and occupation status.

This project is related to two literature strands. In terms of topic, it contributes to recent work on the determinants of consumption expenditure during Covid-19, see for instance Baker et al. (2020) and Chetty et al. (2020) for the US, Chronopoulos et al. (2020) for the UK and Guglielminetti and Rondinelli (2021) and Immordino et al. (2022) for Italy. The organizing framework we use is the Euler equation for consumption which relates expected consumption growth to income expectations and considers income and health risks as sources of consumption risk.

In terms of methodology, we contribute to work that uses subjective expectations of income, employment, retirement income, retirement age, interest rates and other variables which now are widely used by macroeconomists. Manski (2004, 2017) provides an excellent discussion of the advantages of measures of expectations in macroeconomics. The European Central Bank’s (ECB’s) Consumer Expectations Survey (Georgarakos & Kenny, 2020) and the Federal Reserve Bank of New York’s Survey of Consumer Expectations (Armantier et al., 2017) are leading examples of surveys designed to elicit some of these expectations.

The paper is organized as follows. Section 2 reviews consumption studies during the Covid-19 crisis. Section 3 outlines a standard intertemporal model as an organizing framework to estimate the effect of income and health expectations on expected consumption growth. Section 4 describes the data and how we construct the individual distributions of expected growth rates for income and consumption. Section 5 reports the regression results and Section 6 provides some robustness tests. Section 7 concludes.

## Consumption during Covid-19

The Covid-19 crisis was accompanied by a large drop in consumption in both the US and Europe. Understanding the reasons for the extent of the consumption drop, its timing, and its heterogeneity – across both different expenditure categories and different households – has been the focus of much recent research. Most studies rely on retrospective administrative data and do not measure consumer sentiment, income expectations or perceptions of exposure to the pandemic.

There are several possible explanations for the unusually large drop in consumption observed in 2020. First, there was the direct effect of the pandemic and the associated lockdown measures which prohibited several categories of consumption. The drop in consumption was especially large in sectors directly affected by social distancing measures such as accommodation, restaurants, tourism, and transportation. Several studies document that the drop in consumption in the second quarter of 2020 was induced more by these measures than by labor market disruptions.^1^

Second, it is possible that the drop in consumption was due to concern about the risk of infection. Following the initial wave of the pandemic, although consumption was not prohibited, many people chose to reduce their shopping, travel and interactions with friends, relatives, and colleagues due to fear about contracting the virus. While the lockdown effect can be regarded as producing a form of forced saving, the infection-concern effect is a behavioral effect because it was not imposed by lockdown orders. Chetty et al. (2020) find support for this hypothesis by showing that in the US the contraction in spending was more marked for goods and services that require in-person contact. Eichenbaum et al. (2022) show that the consumption impact of Covid-19 is small when people know the true case-fatality rate but large when people have empirically plausible pessimistic prior beliefs about the case-fatality rate. Goolsbee and Syverson (2021) use cell phone data and show that the individual choice to reduce expenditure was more important than the lockdown orders.

A third possible explanation for the drop in consumption is the precautionary saving effect derived from the uncertainty about the length of the health crisis and its potential economic effects on future income, employment prospects and government ability to sustain household budgets through welfare programs. Coibion et al. (2021) use a survey experiment in the ECB Consumer Expectations Survey (CES) and found that macroeconomic uncertainty caused a reduction in consumers’ willingness to spend in the euro area. Christelis et al. (2020) also use CES data and show that the consumption drop was largest among households more fearful that their financial position would deteriorate due to Covid-19. Based on a series of qualitative surveys administered between June 2020 and November 2021, the Bank of Italy suggests that the consumption drop in Italy in 2020-21 and the associated high saving rate were the result of a combination of lockdown and social distancing measures, precautionary reasons, and fear of contagion.^2^

All these studies use retrospective survey or administrative data on consumption. Our approach differs in that we use survey data on consumption expectations in the 12 months following the interview, and study how these expectations are affected by subjective expectations about disposable income, aggregate GDP, various sources of uncertainty and fear of contracting the Covid-19. Therefore, our approach has the potential to identify the drivers of consumption expectations, and possible interventions that might affect them.

Although the paper focuses on the determinants of 2022 consumption expectations, it is also closely related to recent studies that analyze the consumption response in Italy during the pandemic crisis (2020), before the start of the vaccination campaign in January 2021. In a previous study, Immordino et al. (2022) show with survey data that in Italy the probability of consumption drops and increased saving during the pandemic were positively associated to fear of contagion. Using macroeconomic data, Guglielminetti and Rondinelli (2021) find that half of the slump in private consumption in 2020 is explained by the deterioration in economic conditions, and the other half by pandemic-related factors (fear of infection, lockdown policies and increased uncertainty). Their evidence is combined with microdata from the Bank of Italy’s Special Survey of Italian Households, showing an important role of fear of infection and uncertainty about the future. Ercolani et al. (2021) use the same survey in early 2021, and also find an important role for precautionary saving due to higher job uncertainty, perceptions of a protracted health crisis and worries about the risk of a new pandemic occurring in the coming years. Differently from the mentioned studies, as we describe in the next section, our paper uses data available at the end of 2021, when 80% of the population was vaccinated.

## Organizing framework

Our organizing framework is the standard permanent income model with precautionary saving, reviewed in Jappelli and Pistaferri (2017, Chapter 6). With a constant interest rate *r*, the Euler equation for consumption states that the marginal utility of consumption of individual *i* in period *t* is proportional to the expected marginal utility, that is:

1 $$u^\prime \left( {c_{i,t}} \right) = \frac{{1 + r}}{{1 + \delta _{i,t}}}E_{it}u^\prime \left( {c_{i,t + 1}} \right)$$

A second-order Taylor series expansion of $u^\prime (c_{i,t + 1})$ around *c*_*i,t*_ delivers an expression for the expected growth rate of consumption $E_{it}(gc_{i,t + 1})$:

2 $$E_{it}\left( {gc_{i,t + 1}} \right) = \sigma \left( {c_{i,t}} \right)\left( {\frac{{r - \delta _{i,t}}}{{1 + r}}} \right) + \frac{1}{2}p\left( {c_{i,t}} \right)E_{it}\left( {gc_{i,t + 1}^2} \right) + W_{i,t}$$

where $p(c_{i,t}) = - \frac{{u^{\prime \prime \prime }(c_{it})c_{it}}}{{u^\prime\prime (c_{it})}}$ denotes Kimball’s coefficient of relative prudence, *σ*(*c*_*i,t*_) denotes the elasticity of intertemporal substitution, and *W*_*i,t*_ is a remainder term in the Taylor approximation. We assume that the individual discount rate *δ*_*i,t*_ depends on demographic characteristics *X*_*i,t*_. We then obtain a relation between expected consumption growth and expected consumption risk which can be expressed in a regression framework as:

3 $$E_{it}\left( {gc_{i,t + 1}} \right) = \alpha + \beta E_{it}\left( {gc_{i,t + 1}^2} \right) + \gamma ^\prime X_{i,t} + v_{i,t}$$

where *gc*_*i,t*+1_ is the consumption growth of individual *i*, and *v*_*i,t*_ is an error term reflecting higher order terms of the approximation and measurement error.

Notice that the error term in Eq. (3) is not correlated with expected consumption risk. As in Christelis et al. (2020), the use of expectations implies that measurement error which arises from differences between reported and actual expenditures is not relevant in our case since we do not make use of consumption realizations. Therefore, Eq. (3) can be estimated by exploiting cross-sectional variability in expectations about the individual consumption distributions.

Equation (3) assumes that no household is myopic, or liquidity constrained. Let’s now adopt a rule-of-thumb which refers to a situation where for some household consumption equals income (or tracks income closely), and therefore expected consumption growth depends directly on expected income growth. In this way, as is common in the literature, we approximate the behavior of consumers with short horizons, limited resources, or hyperbolic discounting. Accordingly, we augment the regressions as:

4 $$E_{it}\left( {gc_{i,t + 1}} \right) = \alpha + \beta E_{it}\left( {gc_{i,t + 1}^2} \right) + \gamma ^\prime X_{i,t} + \lambda E_{it}\left( {gy_{i,t + 1}} \right) + v_{i,t}$$

where *gy*_*i,t+*1_ is the growth rate of disposable income. The parameter *λ* represents the extent to which expected consumption growth responds to income growth over and above the amount warranted by the permanent income model, that is, the excess sensitivity of consumption growth to expected income growth. One way to interpret this parameter is to posit that each household sets consumption equal to income with probability *λ* (perhaps because of binding liquidity constraints or myopia) and follows the permanent income model with probability (1-*λ*).

To make Eq. (4) operational we proxy consumption risk by the following potential sources of underlying risks relevant during the pandemic crisis: individual income risk, probability of social distancing measures, fear of infection, and expectations on the vaccination rate (or a qualitative indicator of the health crisis). In the regressions, we distinguish also between individual and aggregate expected income growth. In each case, we maintain the hypothesis that these risks are unavoidable and exogenous which of course is debatable, at least in the case of individual income risk, fear of infection and vaccination rates.

Our framework allows us to verify the main factors that potentially affect expected consumption growth: (i) an idiosyncratic component, captured by income risk and the risk of future social distancing measures, (ii) an aggregate component, captured by expectations about GDP growth and health conditions, (iii) an excess sensitivity component, capturing the behavior of rule-of-thumb consumers whose consumption path is closely correlated to their income path.

## The ISEC survey

To study the determinants of the consumption expectations of Italian households during the Covid-19 crisis, we designed the ISEC. We commissioned Doxa, a leading Italian polling agency that is engaged in market research and social studies, to administer the survey. The survey was aimed at eliciting information on expected consumption in 2022, distinguishing between total household consumption, food consumption at home, food consumption away from home and online purchases. We also asked about income expectations both individual and aggregate, that is, expected individual income growth, probability of unemployment (for both the employed and the unemployed), aggregate GDP growth, and pandemic-related perceptions such as individual fear of contagion during economic activities, probability of future lockdowns and a qualitative indicator about the general health situation.

The ISEC survey was administered to a representative sample of the Italian resident population aged between 18 and 75 and included 3016 households. The sampling scheme is like that employed by the Bank of Italy Survey of Household Income and Wealth (SHIW), which is a representative survey of the Italian population. In ISEC the Italian resident population is stratified according to three criteria: geographic area of residence (North-East, North-West, Central, South), age group (18–34, 35–44, 45–54, 55–64, over 65) and gender. The survey was administered in the two weeks between 20 November and 5 December 2021, which were the weeks before the fourth wave of the pandemic.^3^

Table 1 presents the ISEC survey demographic and occupational characteristics for the initial total sample (3,016 observations, column 1) and for the estimation sample (2385 observations, column 2),^4^ and compares them to those of the SHIW 2020 (column 3), the most recent release of the Bank of Italy. The comparison highlights similarities between the two surveys but also features that are specific to ISEC.

**Table 1:** ISEC-SHIW comparison

	ISEC Total sample	ISEC Estimation sample	SHIW 2020
Male	0.49	0.53	0.51
Female	0.51	0.47	0.49
Age			
18–34	0.24	0.26	0.22
35–44	0.17	0.13	0.17
45–54	0.23	0.21	0.21
55–64	0.24	0.22	0.21
65-over	0.12	0.12	0.19
Education			
Primary education	0.19	0.17	0.40
Secondary education	0.55	0.56	0.42
Tertiary education	0.26	0.27	0.18
Sector of activity			
Retired	0.13	0.12	0.16
Not employed	0.14	0.13	0.09
Household size			
1 member	0.09	0.08	0.13
2 members	0.28	0.27	0.26
3 members	0.30	0.31	0.27
4 members	0.26	0.26	0.25
5 or more members	0.07	0.07	0.09
Geographical area			
North	0.46	0.46	0.46
Center	0.20	0.20	0.19
South and Islands	0.34	0.34	0.35
Observations	3016	2385	11,374

Note: The table compares sample means of selected demographic variables in the 2021 ISEC and 2020 Bank of Italy Survey of Household Income and Wealth (SHIW). In the SHIW we consider only households headed by individuals in the 18–75 age range. Sample means are computed using sample weights. “Not employed” includes the unemployed and those looking for a first job

If we compare columns (1) and (3), we observe no appreciable differences between the two surveys for the gender and regional variables. ISEC includes a larger proportion of respondents aged 55 to 64 (3 percentage points higher), and a correspondingly lower proportion of respondents aged over 65 (7 points lower). These age differences reflect the lower proportion of retired individuals in the ISEC. In the ISEC sample, education levels are higher: the proportion of respondents with tertiary education is 26% in ISEC compared to 18% in the SHIW, and the proportion of individuals with secondary education is 55% in ISEC and 42% in the SHIW. The oversampling of individuals with higher education is common in surveys conducted using CAWI methods because more highly educated respondents are more likely to have internet access and therefore are more likely to respond to an online questionnaire. The reader may refer to section B of the Appendix for a detailed description of ISEC and the questionnaire.

To measure expected consumption growth we asked respondents to assign points to each of the following seven scenarios regarding household consumption growth in the 12 months following the survey relative to the previous 12 months:^5^ (i) decrease by more than 10%; (ii) decrease by between 5% and 10%; (iii) decrease by between 0% and 5%; (iv) approximately the same; (v) increase by between 0% and 5%; (vi) increase by between 5% and 10%; (vii) increase by more than 10%. We required the sum of points to equal 100, and then normalized this value to probabilities summing to one. Figure A1 in section A of the Appendix plots the distributions of the probability of consumption growth in 2022 for each of the seven intervals provided to respondents. Using this information, and assuming that household expectations can be approximated by the expectations of the respondent, we calculate for each household the first and second moments of the distributions of expected consumption growth.

**Fig. 1:**
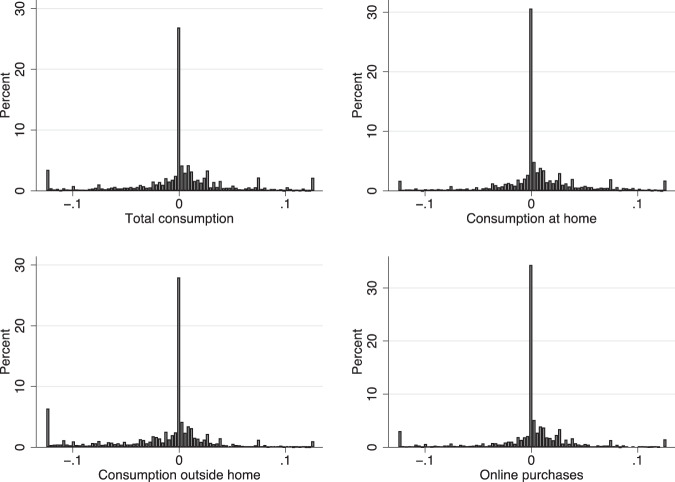
The distribution of expected consumption growth. The figure plots the cross-sectional distribution of expected total consumption growth, consumption, at home, outside home and online purchases

Figure 1 displays the cross-sectional distribution of the expected growth rate of total consumption, food consumption at home, away from home and online purchases. All four distributions behave similarly. The histogram of total consumption shows a mass around 0% consumption growth: about 25% of respondents expected consumption to be “approximately the same” as in the previous 12 months. However, there also is considerable heterogeneity in expectations: 32% are pessimistic (expect negative total consumption growth), while 43% are optimistic (expect positive growth). Among the latter group, 12% expect consumption growth higher than 5%. The average standard deviation of the individual distributions is 0.033, again with considerable heterogeneity: 27% report point expectations (and therefore the standard deviation of the distribution is zero), while for 20% of the sample the standard deviation exceeds 0.06 and for 5% it exceeds 0.083.^6^ Table 2 shows that the only consumption component with average expected negative growth rate is consumption away from home.^7^

**Table 2:** Descriptive statistics

	Mean	Median	Std. Dev
	(1)	(2)	(3)
Expected total consumption growth	0.001	0	0.049
Expected growth of food consumption at home	0.006	0	0.043
Expected growth of food consumption away from home	−0.012	0	0.050
Expected growth of online purchases	0.001	0	0.043
Expected disposable income growth	−0.013	0	0.043
Expected GDP growth	0.023	.02	0.029
Income risk	0.004	.003	0.004
Prob. of lockdown	0.584	.6	0.247
Average fear	0.576	.6	0.245
Expected vaccination rate	0.338	.375	0.158
Age/100	0.468	.48	0.141
Male	0.531	1	0.499
Family size	2.995	3	1.106
College	0.272	0	0.445
High school	0.558	1	0.497
Self-employed	0.104	0	0.305
Unemployed	0.13	0	0.337
Retired	0.116	0	0.320
Not working	0.177	0	0.382
South	0.34	0	0.474
Center	0.202	0	0.401
Most affected sector	0.187	0	0.390
Vaccine (at least one dose)	0.917	1	0.277
Experienced Covid-19 infection	0.091	0	0.288
Probability of unemployment	0.236	.2	0.264
Income (euros)	2364	1750	2251

Note. Summary statistics refer to the ISEC sample of 2385 observations used in the regression analysis. Income risk is the second moment of the distribution of expected income. See the Appendix for the definition of the variables

Using the same format as the questions about consumption expectations, we compute the expected growth rate of disposable income in the 12 months following the survey. On average, respondents expect negative income growth (–1.3%), again with considerable heterogeneity.^8^ In particular, 26% expect no income change, 28% expect positive income growth and 46% expect their income to drop. The average standard deviation of the individual distributions is close to the same statistic for the expected consumption growth distribution (0.032), with 26% of respondents reporting point expectations (no income variability). For 20% of the sample the standard deviation exceeds 0.04, and for 5% it exceeds 0.08.

The ISEC survey also asked respondents to report macroeconomic forecasts of GDP growth rate in 2022. Table 2 shows that average expected GDP growth is 2.3%. The majority of respondents (75%) expected positive GDP growth, and 21% expected growth to be higher than 4%. These aggregate forecasts are lower than the November 2021 forecasts from the Italian government, the Bank of Italy, and international organizations for 2022 (all above 4%). However, they are considerably higher than the forecasts of individual disposable income growth which suggests that respondents are more optimistic about the income of others relative to their own income.

**Fig. 2:**
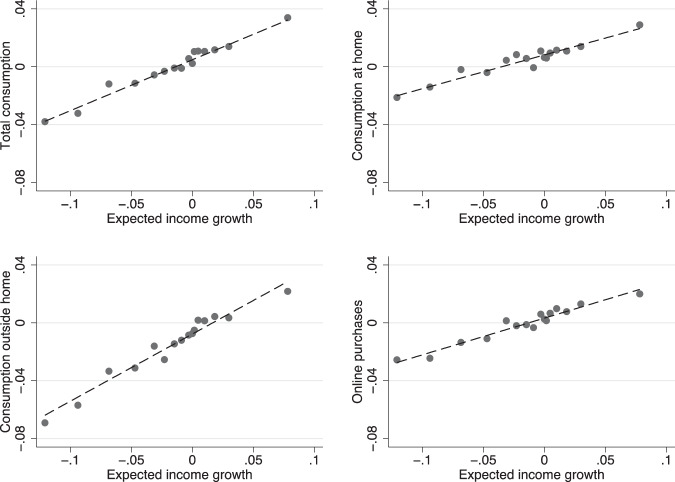
Expected consumption growth and expected income growth. The figure plots expected total consumption growth, consumption, at home, outside home and online purchases against bins of the cross-sectional distribution of expected income growth

Figure 2 plots the four expected consumption growth indicators against the expected disposable income growth (taking averages within each distribution bin). Consumption and income expectations are well aligned, and the slopes are positive in all cases. The correlations are much less than 1 however, and lower in magnitude for food consumption at home and online purchases. Indeed, the slope of the bivariate regression of expected consumption growth on income growth ranges from 0.21 for food consumption at home to 0.47 for food consumption away from home.

**Fig. 3:**
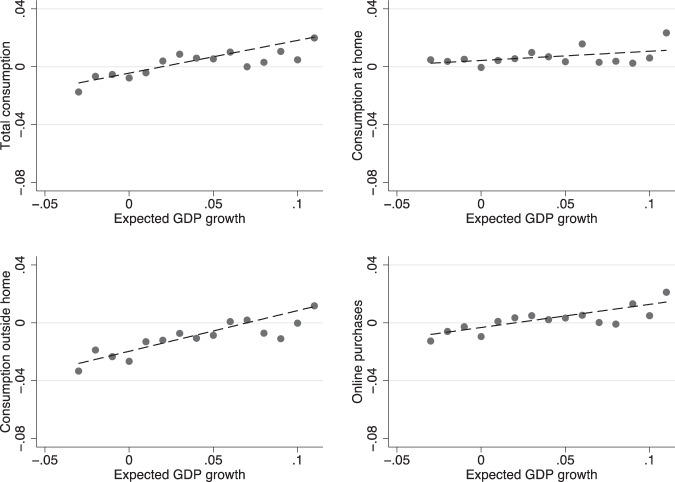
Expected consumption growth and expected GDP growth. The figure plots expected total consumption growth, consumption at home, outside home and online purchases against bins of the cross-sectional distribution of expected GDP growth

Figure 3 plots the same consumption growth indicators against the expected GDP growth. Again, in this case the correlations are all positive but lower than for disposable income growth, particularly for food consumption at home. The slope ranges from 0.06 for food consumption at home to 0.27 for consumption away from home. Taken together, Figs. 2 and 3 suggest that expectations about individual and aggregate income are potentially important drivers of consumption expectations.

One of the objectives of the paper is to explore whether uncertainty affects expected consumption growth, as predicted by models with precautionary saving. We consider income and pandemic-related uncertainties as the two main sources of risk in November 2021. We measure individual income risk by the second moment of the distribution of expected income growth, as described above. We consider also the self-reported probability that respondents will be in their current job in 2022 (if employed in November 2021), or will find a new job (if looking for a job or unemployed).

In the case of pandemic-related risk, we focus on four variables: fear of contagion, probability of future lockdowns, and quantitative and qualitative indicators of the general health situation. Depending on their intensity, each of these variables could potentially limit work and consumption activities associated with precautionary saving. Section B of the Appendix reports the wording of each of the health indicators.

For fear of contagion, we elicit individual perceptions of fear of Covid-19 by asking about the perceived risks associated with three economic and social activities: (i) working, (ii) shopping, eating out or traveling, (iii) contact with relatives or friends. Each of these variables is coded from 1 (not worried), to 10 (extremely worried). We then compute the average of the three variables, normalize it to 1, and label it “average fear”.

**Fig. 4:**
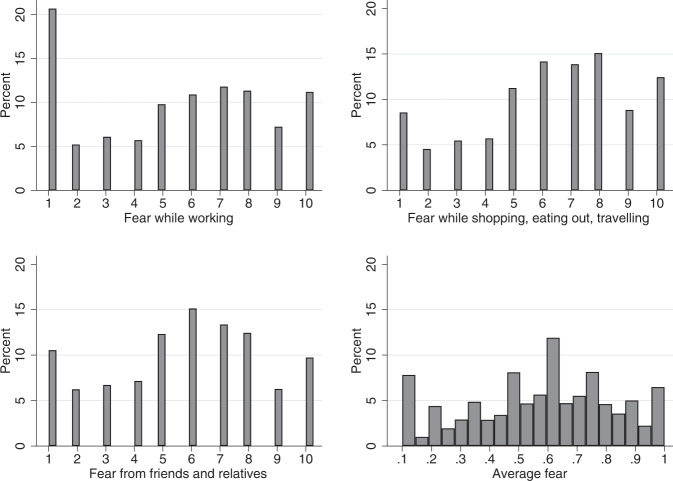
The distribution of Covid-19 fear of contagion. The figure plots the distribution of fear from infection while working, while shopping, eating out or traveling, and fear for infection from friends and relatives. The bottom-left graph plots the average of the three variables, standardized between zero and one

Figure 4 plots the distribution of the three indicators of fear, and of their average. The distribution of fear while shopping is mostly between 6 and 8, while fear of contacts it is between 5 and 7. The distribution of fear related to work also peaks between 6 and 8, while 19.6% report “no fear” (half of these are unemployed or retired). The mean of the normalized measure of “average fear” is 0.58.

The ISEC survey also asked about the probability of future restrictions on social and economic activities such as another lockdown like the one implemented in March and April 2020 or partial lockdowns imposing different constraints.^9^ We asked respondents to rate their expectations on a scale from 1 (extremely unlikely) to 10 (extremely likely). The average for lockdown probability is 0.58 with over 75% of respondents reporting a probability greater than or equal to 50%.

As quantitative indicator of the severity of the pandemic, we consider the expected vaccination rate; that is, the individual perception about the fraction of Italian individuals (aged over 2) who will receive the booster (third dose) by the end of January 2022. As an additional, qualitative indicator, we ask respondents their expectations about the general health situation, with five outcomes, ranging from much worse to much better.

Table 2 presents the summary statistics for the main variables that we use in the regression analysis. In the ISEC estimation sample, 10.4% of respondents are self-employed, 13% are unemployed and 11.6% are retired. Average monthly disposable income is 2360 euros (median 1750 euros). Comparison with SHIW 2020 figures shows that weighted average disposable income is lower (1429 euros, with a median value of 1391 euros). Taken together, these numbers suggests that ISEC includes a slightly higher proportion of working age respondents (18–64), with slightly higher disposable income. Overall, the two samples are aligned across many dimensions, as showed in Table 1.

## Regression results

Table 3 reports the regression results for expected total consumption growth in 2022. The right-hand-side variables in column (1) include only expected growth rate of disposable income, expected GDP growth, second moment of the distribution of expected income growth (our proxy for income risk), probability of lockdown, average fear of contagion and expected vaccination rate.

**Table 3:** Regressions for total consumption

	Baseline	With demographics	Low income	High income
	(1)	(2)	(3)	(4)
Expected disp. income growth	0.331 (0.030)***	0.326 (0.030)***	0.453 (0.053)***	0.234 (0.038)***
Expected GDP growth	0.139 (0.036)***	0.121 (0.036)***	0.108 (0.064)*	0.118 (0.043)***
Income risk	0.104 (0.282)	0.243 (0.285)	−0.063 (0.506)	0.747 (0.350)**
Prob. of lockdown	0.001 (0.004)	0.001 (0.004)	0.010 (0.009)	−0.004 (0.005)
Average fear	−0.005 (0.004)	−0.005 (0.005)	−0.007 (0.009)	−0.003 (0.005)
Expected vaccination rate	0.009 (0.006)	0.007 (0.006)	0.009 (0.012)	0.008 (0.008)
Age/100		0.001 (0.008)	−0.011 (0.017)	0.008 (0.009)
Male		−0.000 (0.002)	−0.005 (0.004)	0.001 (0.002)
Family size		−0.000 (0.001)	0.001 (0.002)	−0.002 (0.001)
College		0.005 (0.003)*	0.001 (0.006)	0.008 (0.004)*
High school		0.002 (0.003)	−0.000 (0.005)	0.004 (0.003)
Self-employed		−0.006 (0.003)*	−0.009 (0.006)	−0.002 (0.004)
Unemployed		−0.006 (0.003)*	−0.006 (0.005)	−0.003 (0.004)
Retired		0.002 (0.004)	0.011 (0.008)	−0.002 (0.004)
Not working		−0.003 (0.003)	0.000 (0.005)	−0.005 (0.003)
South		0.001 (0.002)	0.003 (0.004)	−0.000 (0.003)
Center		−0.001 (0.003)	0.006 (0.006)	−0.004 (0.003)
Log income		0.006 (0.001)***	0.004 (0.002)***	0.004 (0.002)*
Constant	0.001 (0.004)	−0.044 (0.011)***	−0.033 (0.017)*	−0.030 (0.020)
*R*^2^	0.10	0.11	0.17	0.07
*N*	2385	2385	796	1589

Note. The table reports OLS estimates with robust standard errors. Low and high income refer, respectively, to households with disposable income below and above the median. Standard errors are reported in parenthesis. ***, **, and * indicate statistical significance at the 1%, 5% and 10% confidence level, respectively

The results show that respondents’ expectations about disposable income are the most important drivers of expected consumption growth. The coefficient is quite precisely estimated, showing that a 1 percentage point increase in expected disposable income growth is associated with 0.33 percentage points higher expected consumption growth. This result could be interpreted as each household setting its consumption growth equal to its expected income growth with a 0.3 probability; alternatively, that about one third of households are hand-to-mouth households whose expected consumption path is closely aligned to their income path.^10^ The results also suggest significant sensitivity of expected consumption growth with respect to aggregate GDP growth. The estimated coefficient suggests that a 1 percentage point increase in GDP growth is associated with 0.14 percentage points of expected consumption growth. In this specification, the coefficients of income risk, average fear and expected vaccination rate are not statistically different from zero.^11^

It is interesting to compare our results with the evidence for Italy provided by Guglielminetti and Rondinelli (2021), Ercolani et al. (2021) and Immordino et al. (2022). Using data up to February 2021, these papers show that the drop in consumption during the pandemic is associated also to health-related variables (infection fear, perceptions of a protracted health crisis and worries about the risk of a new pandemic). ISEC instead was fielded in November 2021, when approximately 80% of the population was vaccinated and the general economic situation improved substantially with respect the start of the year.^12^ Another important difference with previous studies is that Guglielminetti and Rondinelli (2021) and Immordino et al. (2022) use a dummy for the expected consumption decline and a reduced form approach, while in the present paper we use a continuous variable for expected consumption growth and an approximation of the first order condition of consumption optimization. This allows us to provide a structural interpretation of some of the estimated parameters, using as benchmark the permanent income model with income risk.

Table 3 column (2) extends the baseline specification by including the demographic variables (age/100, gender, family size, high-school and college dummies, employment, and regional dummies) and the log of disposable income in 2021. The estimated coefficients of expected disposable income growth and aggregate GDP growth rate are not affected. The coefficient of log income is positive and significant, unemployed individuals relative to other individuals expect lower consumption growth (coefficient is –0.6%) and the same is true for self-employed (–0.6%) while individuals with a college degree display higher growth (0.5%). The other coefficients are not statistically different from zero.

In columns (3) and (4) the sample is split into two groups. Column (3) includes only households with current income strictly below the median (1,750 euros); in column (4) the sample includes individuals whose income is equal to or above the median. The sensitivity of expected total consumption growth to expected GDP growth does not differ between the two groups. However, the coefficient of expected disposable income growth is almost twice as large for the low-income group (0.453 vs. 0.234). Both coefficients are precisely estimated, and are also statistically different from each other. Furthermore, the coefficient of income risk is positive and statistically different from zero only for the high-income group. These findings are broadly in line with the hypothesis that liquidity constraints and/or myopic behavior are more prevalent in the low-income group, and that precautionary saving considerations are more important among high-income households.

Tables 4–6 report similar regressions for expected food consumption growth at home, away from home and online, respectively. In the regressions for consumption growth at home (Table 4) the coefficient of expected income growth is lower (0.22) than the coefficient of total consumption and is not statistically different between low and high-income households. Also, expected GDP growth does not predict expected consumption at home. These results suggest that the basket of goods consumed at home, which includes mostly necessity goods, is less sensitive to individual and aggregate expected income growth. Moreover, the expected vaccination rate predicts expected consumption at home especially for the low-income individuals (coefficient is 2%).

**Table 4:** Regressions for food consumption at home

	Baseline	With demographics	Low income	High income
	(1)	(2)	(3)	(4)
Expected disp. income growth	0.224 (0.030)***	0.225 (0.030)***	0.279 (0.054)***	0.190 (0.037)***
Expected GDP growth	0.012 (0.032)	0.009 (0.032)	−0.019 (0.058)	0.023 (0.038)
Income risk	0.138 (0.279)	0.265 (0.281)	0.295 (0.512)	0.374 (0.337)
Prob. of lockdown	−0.002 (0.004)	−0.002 (0.004)	−0.003 (0.008)	−0.002 (0.004)
Average fear	−0.003 (0.004)	−0.001 (0.004)	−0.004 (0.008)	0.000 (0.004)
Expected vaccination rate	0.012 (0.006)**	0.010 (0.006)*	0.022 (0.012)*	0.004 (0.007)
Age/100		−0.002 (0.008)	0.002 (0.017)	−0.004 (0.008)
Male		−0.002 (0.002)	−0.003 (0.004)	−0.002 (0.002)
Family size		0.001 (0.001)	0.003 (0.002)**	−0.000 (0.001)
College		−0.002 (0.003)	0.002 (0.006)	−0.005 (0.003)
High school		0.000 (0.003)	0.003 (0.005)	−0.001 (0.003)
Self-employed		−0.005 (0.003)*	−0.011 (0.006)*	−0.001 (0.003)
Unemployed		−0.008 (0.003)***	−0.009 (0.005)*	−0.007 (0.003)**
Retired		0.003 (0.003)	0.007 (0.007)	0.001 (0.003)
Not working		−0.003 (0.002)	−0.002 (0.005)	−0.004 (0.003)
South		−0.003 (0.002)	−0.006 (0.004)	−0.002 (0.002)
Center		−0.002 (0.002)	−0.008 (0.005)	0.000 (0.003)
Log income		0.002 (0.001)	0.001 (0.002)	0.002 (0.002)
Constant	0.007 (0.004)*	−0.008 (0.012)	−0.008 (0.021)	0.003 (0.018)
*R*^2^	0.05	0.06	0.08	0.04
*N*	2385	2385	796	1589

Note. The table reports OLS estimates with robust standard errors. Low and high income refer, respectively, to households with disposable income below and above the median. Standard errors are reported in parenthesis. ***, **, and * indicate statistical significance at the 1%, 5% and 10% confidence level, respectively

**Table 5:** Regressions for food consumption away from home

	Baseline	With demographics	Low income	High income
	(1)	(2)	(3)	(4)
Expected disp. income growth	0.444 (0.030)***	0.428 (0.030)***	0.549 (0.049)***	0.353 (0.039)***
Expected GDP growth	0.150 (0.035)***	0.150 (0.035)***	0.085 (0.062)	0.171 (0.043)***
Income risk	0.142 (0.280)	0.155 (0.284)	0.872 (0.472)*	0.015 (0.356)
Prob. of lockdown	−0.009 (0.004)*	−0.008 (0.004)*	−0.008 (0.008)	−0.007 (0.005)
Average fear	0.001 (0.004)	−0.001 (0.005)	−0.001 (0.008)	0.001 (0.006)
Expected vaccination rate	−0.007 (0.006)	−0.005 (0.006)	−0.014 (0.011)	−0.001 (0.008)
Age/100		−0.019 (0.008)**	−0.062 (0.017)***	0.001 (0.009)
Male		0.002 (0.002)	0.002 (0.004)	0.000 (0.002)
Family size		0.000 (0.001)	−0.000 (0.002)	−0.001 (0.001)
College		0.002 (0.003)	−0.003 (0.006)	0.002 (0.004)
High school		−0.001 (0.003)	−0.003 (0.005)	−0.001 (0.003)
Self-employed		−0.005 (0.003)	−0.013 (0.006)**	0.000 (0.004)
Unemployed		−0.011 (0.003)***	−0.015 (0.005)***	−0.006 (0.004)
Retired		−0.005 (0.004)	−0.004 (0.008)	−0.006 (0.004)
Not working		−0.004 (0.003)	−0.003 (0.005)	−0.004 (0.003)
South		−0.004 (0.002)*	−0.003 (0.004)	−0.003 (0.003)
Center		−0.004 (0.003)	−0.001 (0.005)	−0.005 (0.003)*
Log income		0.003 (0.002)*	−0.002 (0.003)	0.003 (0.003)
Constant	−0.004 (0.004)	−0.015 (0.014)	0.043 (0.023)*	−0.025 (0.022)
*R*^2^	0.16	0.17	0.24	0.12
*N*	2385	2385	796	1589

Note. The table reports OLS estimates with robust standard errors. Low and high income refer, respectively, to households with disposable income below and above the median. Standard errors are reported in parenthesis. ***, **, and * indicate statistical significance at the 1%, 5% and 10% confidence level, respectively

**Table 6:** Regressions for online purchases

	Baseline	With demographics	Low income	High income
	(1)	(2)	(3)	(4)
Expected disp. income growth	0.258 (0.029)***	0.246 (0.028)***	0.336 (0.049)***	0.184 (0.035)***
Expected GDP growth	0.096 (0.031)***	0.093 (0.032)***	0.080 (0.055)	0.096 (0.038)**
Income risk	0.267 (0.263)	0.333 (0.263)	0.479 (0.468)	0.414 (0.311)
Prob. of lockdown	0.003 (0.004)	0.003 (0.004)	0.009 (0.008)	0.000 (0.004)
Average fear	0.000 (0.004)	−0.000 (0.004)	−0.003 (0.008)	0.002 (0.005)
Expected vaccination rate	0.005 (0.006)	0.005 (0.006)	0.002 (0.011)	0.007 (0.007)
Age/100		−0.022 (0.008)***	−0.033 (0.016)**	−0.015 (0.009)*
Male		0.000 (0.002)	0.002 (0.004)	−0.001 (0.002)
Family size		−0.000 (0.001)	−0.002 (0.001)	0.000 (0.001)
College		0.005 (0.003)*	0.008 (0.006)	0.003 (0.003)
High school		0.006 (0.003)**	0.010 (0.005)**	0.004 (0.003)
Self-employed		−0.006 (0.003)*	−0.007 (0.006)	−0.004 (0.003)
Unemployed		−0.009 (0.003)***	−0.012 (0.005)**	−0.003 (0.003)
Retired		0.004 (0.003)	0.006 (0.006)	0.003 (0.003)
Not working		−0.000 (0.002)	0.006 (0.005)	−0.004 (0.003)
South		−0.002 (0.002)	−0.003 (0.004)	−0.000 (0.002)
Center		−0.003 (0.002)	0.001 (0.005)	−0.005 (0.003)*
Log income		0.003 (0.002)**	0.001 (0.003)	0.003 (0.002)
Constant	−0.002 (0.003)	−0.018 (0.012)	0.001 (0.022)	−0.020 (0.018)
*R*^2^	0.07	0.09	0.14	0.05
*N*	2385	2385	796	1589

Note. The table reports OLS estimates with robust standard errors. Low and high income refer, respectively, to households with disposable income below and above the median. Standard errors are reported in parenthesis. ***, **, and * indicate statistical significance at the 1%, 5% and 10% confidence level, respectively

The regressions for expected consumption away from home (Table 5) are aligned to total consumption (Table 3). The coefficient of expected income growth is 0.44 and is larger for the low-income group (0.55) relative to the high-income group (0.35). The coefficient of expected GDP growth is around 0.15 and is statistically different from zero in the total and the high-income samples. In columns (1) and (2) the probability of lockdown is negatively associated with expected consumption growth: going from the lowest to the highest reported lockdown probabilities, reduces expected consumption growth by 1 percentage point, which could be expected given that lockdowns reduce social interactions, recreational activities, shopping and restaurant visits. In contrast with the results of Table 3, income risk is (weakly) significant for low-income individuals for consumption away from home, but not for high-income individuals. The results might indicate that our test for precautionary saving has relatively low power, but note also that the implications of models with precautionary saving for total consumption do not mechanically translate into predictions about components of consumption (such as food at home, away from home or online purchases).

Table 6 uses expected growth in online purchases as the dependent variable. Again, the results are in line with our findings for total consumption. It is worth noting that the probability of lockdown and other health-related variables are not associated with expectations of online purchases. In contrast, European Central Bank ECB (2022) finds that cashless means of payment are becoming increasingly important in the euro area and that the trend towards cashless payments seems to have accelerated during the pandemic.^13^ Our guess is that at the end of 2021 health-related factors were no longer significant in explaining expectations about the dynamics of online purchases in 2022. Notice however that we cannot rule out measurement error (our variables are not a good proxy for the importance of card-based transaction), or collinearity with other regressors.

## Robustness checks

In this section we report various exercises to check the reliability of our sample to the presence of heaping and missing values, and the robustness of our specification.

The literature on subjective expectations refers repeatedly to individual answers tending to heap around certain values. This might be the result of rounding which could complicate the analysis because the rounding might be done at different levels; some rounding might be done at multiples of 10 or 5, others might focus on extreme values such as 0, 50 and 100.^14^ To investigate the degree of heaping, Figures A1 and A2 in the Appendix provide histograms of the probabilities assigned to expected total consumption and income growth intervals in the seven intervals specified in the questionnaire.

We observe that the probabilities vary with the income and consumption intervals (generally lower for intervals that include more extreme values for consumption and income growth) but are not clustered around the same values. We observe no indication of the prevalence of “50% responses” in any of the intervals.

To check this in more detail, we examine whether rounding was correlated to household characteristics. In line with Manski and Molinari (2010), we notice that people tend to report probabilities in multiples of 5 and 10 (M5 and M10), and estimate probit regressions for the probability of reporting these values. Tables A1 and A2 in the Appendix report the regressions for M10 for expected income and consumption growth; the regressions for M5 are similar. Except for the central interval of expected income and consumption growth (young males tend to round less), there is no systematic evidence of significant rounding according to age, gender, income, or education.

In the survey respondents assign probability weights to seven scenarios. While six of them are quantitatively defined (e.g., “decrease by between 0% and 5%”), one of them is qualitative (“approximately the same”) and does refer to a value around 0%. In coding the expectation questions about consumption and income, we assign a value equal to 0 to the qualitative response. To address this issue, we check the robustness of our coding, and find no appreciable change of the results (available upon request).^15^

Item non-responses are another potential threat to the reliability of our estimates. Four variables included in the estimations contain a significant number of non-responses. We note that if we drop expected GDP growth, expected vaccination rate, average fear and probability of lockdown, the results for the other variables are largely unaffected (column (1) in Table A3 in the Appendix). Next, we impute missing values for these four variables using a multiple imputation method with five replicates, based on the same set of regressors as in the baseline regression. Table A3 column (2) in the Appendix reports the results for the baseline specification using these imputed values for the full sample of 3,016 observations; they confirm the initial results.

A second set of checks is about the stability of our baseline specification. Using the whole sample and interacting expected income growth and expected GDP growth with an income dummy provides an alternative way to test for differences in expected income growth responses between low and high-income households. The regression, reported in Table A3 column (3) in the Appendix, confirms our results.

Fear of contagion is higher, on average, in Southern Italy although during the first wave of the epidemic, Northern Italy had a higher number of Covid-19 cases. Initially lockdown measures were uniform across all Italian regions; from November 2020 they differ by region depending on the prevalence of Covid-19 cases. Therefore, our Covid-19-related regressors might be correlated with regional effects. Also, omitted regional effects might be correlated with expected income growth. The regression in column (4) of Table A3 in the Appendix includes regional fixed effects. Again, our initial results are confirmed.^16^

Next, we use additional indicators based on two questions asking if the general health or economic situations will be, in 2022 relative to 2021, much worse, slightly worse, more or less the same, slightly better, or much better. We also consider a dummy for experiencing Covid-19, indicating that in November 2021, 9% of respondents reported that they have been infected with Covid-19, and a dummy for having taken at least one dose of vaccine in November 2021 (91% of respondents). Results in columns (1) and (2) of Table A4 in the Appendix for total consumption growth indicate no association between the qualitative health and GDP indicators and Covid-19 infection. Vaccination instead is positively associated with consumption growth (but notice that vaccination is clearly a choice variable).

Since the pandemic had stronger effects in sectors most affected by lockdown and social distancing policies implemented in 2020-21 (travel, recreational and personal services, restaurants, transports, etc.), it is interesting to check if consumption expectations for 2022 differ by sectors. Our survey has only a coarse sector definition, and accordingly we classify as “most affected sectors” the Construction, Retail and Transportation sectors, and as “less affected” all other sectors. We split the sample according to this classification, also including individuals out of the labor force in the “less affected sectors”. The last two columns of Table A4 in the Appendix show that the coefficients of expected disposable income and GDP growth are larger in the most affected sectors, while other coefficients are similar to the whole sample estimates.

## Summary

We analyze the determinants of expected consumption growth in a sample of over 3000 Italian households interviewed in November 2021, at the end of the first year of the vaccination campaign. Our survey elicited individual distributions for expected consumption growth (total, food at home, away from home, online purchases), expected income growth (individual and aggregate) and pandemic-related variables (probability of lockdown, fear of contagion and expected vaccination rate).

We employ a standard intertemporal consumption model as our organizing framework and show that expected household income and aggregate GDP growth are associated strongly with expected consumption growth. An intuitive interpretation of this result is that for around a third of households, consumption tracks income closely, and that around half of the households in the relatively low-income group are more likely to be credit constrained or have short horizons. Regressions for total consumption indicate also that expected consumption growth is positively associated with expected income risk, particularly for individuals with relatively high incomes, as predicted by models with precautionary saving; however, the association is weaker and statistically insignificant for consumption components.

We also find that health-related variables (quantitative and qualitative forecasts about the health crisis, expectations of future lockdowns, vaccination rates) are not drivers of consumption expectations for 2022. This result contrasts with evidence on consumption during the pandemic, showing that before the vaccination campaign took off, fear of contagion and lockdown policies were negatively associated with consumption (Guglielminetti and Rondinelli, 2021; Immordino et al., 2022). Our survey was fielded in November 2021, when approximately 80% of the population was vaccinated and the general health situations improved substantially with respect to the peak of the pandemic period, explaining why the impact of health-related concerns on consumption was attenuated with respect to the previous year.

## Supplementary Information

Supplementary Information

## Footnotes

1 See Baker et al. (2020) and Cox et al. (2020) for the US, Chronopoulos et al. (2020), Dunn et al. (2021) and Hacioglu et al. (2021) for the UK, Andersen et al. (2022) for Denmark, Bounie et al. (2023) for France and Carvalho et al. (2021, 2022) for Portugal and Spain.

2 See Rondinelli and Zanichelli (2021) for the latest survey, and Bank of Italy, Economic Bulletin, January 2021.

3 The ISEC sample is drawn from a larger representative sample of 120,000 individuals maintained and updated regularly by Doxa. All the interviews were enabled by a Computer Assisted Web Interviewing (CAWI) method. The overall response rate was 71.2%, with low unit non-response for all questions. The questionnaire was constructed in October 2021 by the paper’s authors with the help of field experts. A pilot survey of 100 interviews was conducted in the last week of October 2021, and the questionnaire was revised in early November 2021.

4 The estimation sample is the sample used in the empirical analysis. It excludes observations with missing values for the following variables: expected GDP growth, probability of lockdown, average fear of contagion and expected vaccination rate. As a robustness check, we replicated the regressions imputing missing values for these variables (Section 6).

5 Since the survey was in conducted at the end of November 2021, expectations refer mostly to 2022.

6 Notice that in the text we report the average standard deviation of the individual distributions of consumption growth; additionally, Table 2 reports the standard deviation of the distribution of average expected consumption growth.

7 Notice that in Table 2 sample statistics are computed for our regression estimation sample, while column (1) of Table 1 reports the summary statistics for the entire initial sample of all observations.

8 These statistics are broadly comparable to the Bank of Italy survey administered in November 2021 which elicited qualitative indicators of consumption and income expectations (Rondinelli & Zanichelli, 2021).

9 On November 4^th^, 2021, the Italian government implemented lockdown measures based on different strictness of social distancing measures, and designated regions as “red,” “orange,” “yellow” or “white” depending on the number of their Covid-19 cases, forecasts of the spread of the infection and available intensive care beds.

10 The number is comparable with evidence using different approaches for the prevalence of hand-to-mouth consumption behavior in Italy reviewed in Jappelli and Pistaferri (2017).

11 We also run a robustness check, considering “maximum fear” rather than “average fear”, where “maximum fear” is the maximum of the perceived risks associated with three economic and social activities: (i) working, (ii) shopping, eating out or traveling, (iii) contact with relatives or friends. Results are unchanged.

12 Guglielminetti and Rondinelli (2021) use data from the Bank of Italy’s Special Survey of Italian Households, fielded between August and October 2020, while Immordino et al. (2022) use a survey fielded in October 2020. Ercolani et al. (2021) also use the Bank of Italy’s Special Survey, which was fielded in February and March 2021, two months after the start of the vaccination campaign (27 December 2020), when only about 5% of Italians were vaccinated.

13 Ardizzi et al. (2020) find a substitution effect from cash to card-based transactions during the Covid-19 pandemic.

14 A related issue is that values such as 50 might reflect a form of ignorance or be interpreted as a symptom of respondent uncertainty (Bruine de Bruin et al., 2002). Giustinelli et al. (2022) show that rounding and heaping are associated with observable respondent characteristics such as personal finances, health and macroeconomic events.

15 We replace “decrease by between 0% and 5%” with “decrease by between 1% and 5%” and, similarly, replacing “increase by between 0% and 5%” with “increase by between 1% and 5%”, and consequently replace “approximately the same” with a range of values between –1% to 1% (in further checks, we also experiment with 2–5% and 3–5%). We then assign to each interval the mid-point of the given interval and then compute the moments of the distributions using probabilities in the standard way. We use the new measures of expected consumption growth as our dependent variable, and compute again the first and second moment of expected income growth.

16 As an additional check, we control for possible non-linear effects of income by replacing log income with 15 income-group dummies; again, the estimates are unchanged (results available upon request).
