# Supplementary material for: Consumption and income expectations during Covid-19
Source: Rev Econ Househ. 2023 Apr 22:1–22. Online ahead of print. doi: 10.1007/s11150-023-09656-8 (PMC10122090; doi:10.1007/s11150-023-09656-8)
Supplement: Supplementary file 1 — Supplementary Information [file 11150_2023_9656_MOESM1_ESM.docx]

**Consumption and Income Expectations during Covid-19**

**APPENDIX**

**A. Additional Figures and Tables**

**Figure A1. Probability of expected consumption growth**

Note. The figure plots for each of the intervals of expected consumption growth, the points (standardized in the 0-1 interval) assigned by respondents.

**Figure A2. Probability of expected income growth**

Note. The figure plots for each of the intervals of expected income growth, the points (standardized in the 0-1 interval) assigned by respondents.

**Table A1 – Expected income growth regressions for M10**

|  | Drop more than 10% | Drop  btw 5-10% | Drop btw  0-5% | Unchanged | Increase btw  0-5% | Increase btw  5-10% | Increase more than 10% |
| --- | --- | --- | --- | --- | --- | --- | --- |
|  | (1) | (2) | (3) | (4) | (5) | (6) | (7) |
|  |  |  |  |  |  |  |  |
| Age | -0.0001 | -0.0003 | 0.0001 | 0.0037 | -0.0001 | -0.0001 | -0.0001 |
|  | (0.0003) | (0.0002)** | (0.0002) | (0.0006)*** | (0.0002) | (0.0001) | (0.0001) |
| Male | -0.0130 | -0.0003 | -0.0054 | -0.0464 | 0.0022 | 0.0024 | 0.0054 |
|  | (0.0086) | (0.0045) | (0.0057) | (0.0168)*** | (0.0055) | (0.0027) | (0.0041) |
| College | -0.0072 | -0.0072 | 0.0104 | -0.0013 | 0.0044 | -0.0031 | -0.0014 |
|  | (0.0099) | (0.0048) | (0.0075) | (0.0200) | (0.0068) | (0.0028) | (0.0045) |
| Log income | -0.0195 | 0.0003 | -0.0045 | -0.0069 | 0.0130 | 0.0023 | 0.0036 |
|  | (0.0086)** | (0.0041) | (0.0049) | (0.0145) | (0.0042)*** | (0.0026) | (0.0035) |
|  |  |  |  |  |  |  |  |
| N | 3,016 | 3,016 | 3,016 | 3,016 | 3,016 | 3,016 | 3,016 |
| Pseudo-R2 | 0.0082 | 0.0085 | 0.0054 | 0.0122 | 0.0146 | 0.0169 | 0.0093 |

Note. M10 indicates that probabilities are reported in multiples of 10. The table reports marginal effects from probit estimates with robust standard errors. Standard errors are reported in parenthesis. ***, **, and * indicate statistical significance at the 1%, 5% and 10% confidence level, respectively.

**Table A2 – Expected consumption growth regressions for M10**

|  | Drop  more than 10% | Drop  btw  5-10% | Drop btw  0-5% | Unchanged | Increase btw  0-5% | Increase btw  5-10% | Increase more than 10% |
| --- | --- | --- | --- | --- | --- | --- | --- |
|  | (1) | (2) | (3) | (4) | (5) | (6) | (7) |
|  |  |  |  |  |  |  |  |
| Age | -0.0002 | -0.0001 | 0.0002 | 0.0021 | -0.0001 | 0.0003 | -0.0000 |
|  | (0.0003) | (0.0002) | (0.0002) | (0.0006)*** | (0.0002) | (0.0002) | (0.0002) |
| Male | -0.0118 | -0.0012 | -0.0042 | -0.0568 | 0.0090 | -0.0005 | -0.0047 |
|  | (0.0086) | (0.0044) | (0.0042) | (0.0164)*** | (0.0057) | (0.0058) | (0.0067) |
| College | -0.0082 | -0.0043 | 0.0017 | 0.0019 | 0.0106 | 0.0035 | 0.0054 |
|  | (0.0099) | (0.0050) | (0.0052) | (0.0196) | (0.0077) | (0.0071) | (0.0079) |
| Log income | -0.0320 | -0.0132 | 0.0020 | 0.0013 | 0.0076 | 0.0070 | -0.0020 |
|  | (0.0095)*** | (0.0039)*** | (0.0036) | (0.0141) | (0.0044)* | (0.0046) | (0.0056) |
|  |  |  |  |  |  |  |  |
| N | 3,016 | 3,016 | 3,016 | 3,016 | 3,016 | 3,016 | 3,016 |
| Pseudo-R2 | 0.0179 | 0.0230 | 0.0061 | 0.0064 | 0.0121 | 0.0069 | 0.0013 |

Note. M10 indicates that probabilities are reported in multiples of 10. The table reports marginal effects from probit estimates with robust standard errors. Standard errors are reported in parenthesis. ***, **, and * indicate statistical significance at the 1%, 5% and 10% confidence level, respectively.

**Table A3. Robustness checks for total consumption**

|  | Total ISEC sample  (1) | Imputed values  (2) | Interaction terms  (3) | Region fixed effects  (4) |
| --- | --- | --- | --- | --- |
| Expected disposable income growth | 0.342 | 0.335 | 0.228 | 0.323 |
|  | (0.029)*** | (0.029)*** | (0.038)*** | (0.030)*** |
| Expected GDP growth |  | 0.114 | 0.124 | 0.119 |
|  |  | (0.031)*** | (0.040)*** | (0.036)*** |
| Income risk | 0.055 | 0.099 | 0.453 | 0.245 |
|  | (0.271) | (0.270) | (0.288) | (0.285) |
| Probability. of lockdown |  | -0.000 | 0.001 | 0.001 |
|  |  | (0.004) | (0.004) | (0.004) |
| Average fear |  | -0.003 | -0.005 | -0.005 |
|  |  | (0.004) | (0.005) | (0.005) |
| Expected vaccination rate |  | 0.007 | 0.009 | 0.007 |
|  |  | (0.006) | (0.006) | (0.006) |
| Age / 100 | 0.001 | -0.002 | 0.002 | 0.002 |
|  | (0.007) | (0.007) | (0.008) | (0.008) |
| Male | 0.001 | 0.000 | -0.001 | -0.000 |
|  | (0.002) | (0.002) | (0.002) | (0.002) |
| Family size | 0.001 | 0.001 | -0.000 | -0.000 |
|  | (0.001) | (0.001) | (0.001) | (0.001) |
| College | 0.006 | 0.006 | 0.005 | 0.006 |
|  | (0.003)** | (0.003)** | (0.003)* | (0.003)* |
| High school | 0.002 | 0.002 | 0.003 | 0.003 |
|  | (0.002) | (0.002) | (0.003) | (0.003) |
| Self-employed | -0.004 | -0.005 | -0.005 | -0.006 |
|  | (0.003) | (0.003) | (0.003) | (0.003)* |
| Unemployed | -0.007 | -0.007 | -0.005 | -0.006 |
|  | (0.003)** | (0.003)** | (0.003) | (0.003)* |
| Retired | 0.003 | 0.002 | 0.002 | 0.001 |
|  | (0.003) | (0.003) | (0.004) | (0.004) |
| Not working | -0.003 | -0.003 | -0.002 | -0.002 |
|  | (0.002) | (0.002) | (0.003) | (0.003) |
| South | 0.001 | 0.001 | 0.002 |  |
|  | (0.002) | (0.002) | (0.002) |  |
| Centre | -0.002 | -0.002 | -0.001 |  |
|  | (0.002) | (0.002) | (0.003) |  |
| Log income | 0.005 | 0.004 | 0.004 | 0.006 |
|  | (0.001)*** | (0.001)*** | (0.001)*** | (0.001)*** |
| Exp. Income growth × Low income |  |  | 0.257 |  |
|  |  |  | (0.062)*** |  |
| Exp. GDP growth × Low income |  |  | -0.020 |  |
|  |  |  | (0.059) |  |
| Constant | -0.037 | -0.035 | -0.032 | -0.041 |
|  | (0.011)*** | (0.011)*** | (0.012)*** | (0.019)** |
|  |  |  |  |  |
| *R*^2^ | 0.11 | 0.11 | 0.12 | 0.12 |
| *N* | 3,016 | 3,016 | 2,385 | 2,385 |

Note. The table reports OLS estimates with robust standard errors. Standard errors are reported in parenthesis. ***, **, and * indicate statistical significance at the 1%, 5% and 10% confidence level, respectively.

**Table A4. Robustness checks for total consumption**

|  | Qualitative forecast  (1) | Covid-19 experience  (2) | Most affected sectors  (3) | Less affected sectors  (4) |
| --- | --- | --- | --- | --- |
| Expected disposable income growth | 0.325 | 0.319 | 0.378 | 0.310 |
|  | (0.031)*** | (0.031)*** | (0.065)*** | (0.034)*** |
| Expected GDP growth | 0.102 | 0.121 | 0.125 | 0.121 |
|  | (0.038)*** | (0.036)*** | (0.074)* | (0.041)*** |
| Income risk | 0.267 | 0.199 | 0.539 | 0.193 |
|  | (0.292) | (0.291) | (0.660) | (0.315) |
| Probability. of lockdown | 0.002 | 0.002 | -0.010 | 0.004 |
|  | (0.005) | (0.005) | (0.011) | (0.005) |
| Average fear | -0.006 | -0.009 | -0.012 | -0.004 |
|  | (0.005) | (0.005)* | (0.012) | (0.005) |
| Expected vaccination rate | 0.005 | 0.005 | 0.007 | 0.007 |
|  | (0.007) | (0.007) | (0.015) | (0.007) |
| Age / 100 | 0.001 | -0.002 | 0.003 | -0.001 |
|  | (0.008) | (0.008) | (0.017) | (0.010) |
| Male | -0.001 | -0.001 | -0.001 | -0.001 |
|  | (0.002) | (0.002) | (0.005) | (0.002) |
| Family size | -0.000 | -0.000 | 0.000 | -0.000 |
|  | (0.001) | (0.001) | (0.002) | (0.001) |
| College | 0.005 | 0.005 | -0.003 | 0.006 |
|  | (0.003) | (0.003) | (0.007) | (0.004) |
| High school | 0.002 | 0.002 | -0.002 | 0.003 |
|  | (0.003) | (0.003) | (0.006) | (0.003) |
| Self-employed | -0.006 | -0.005 | -0.012 | -0.002 |
|  | (0.003)* | (0.003) | (0.006)** | (0.004) |
| Unemployed | -0.005 | -0.006 |  | -0.006 |
|  | (0.003) | (0.003)** |  | (0.003)* |
| Retired | 0.002 | 0.002 |  | 0.002 |
|  | (0.004) | (0.004) |  | (0.004) |
| Not working | -0.002 | -0.004 |  | -0.003 |
|  | (0.003) | (0.003) |  | (0.003) |
| South | 0.001 | 0.001 | 0.011 | -0.001 |
|  | (0.002) | (0.002) | (0.005)* | (0.002) |
| Centre | -0.001 | -0.000 | -0.002 | -0.001 |
|  | (0.003) | (0.003) | (0.007) | (0.003) |
| Log income | 0.006 | 0.005 | 0.009 | 0.006 |
|  | (0.001)*** | (0.001)*** | (0.004)** | (0.001)*** |
| Qualitative health forecast | 0.076 |  |  |  |
|  | (0.137) |  |  |  |
| Qualitative macro forecast | 0.004 |  |  |  |
|  | (0.014) |  |  |  |
| Vaccine (at least one dose) |  | 0.012 |  |  |
|  |  | (0.004)*** |  |  |
| Experienced Covid-19 |  | -0.000 |  |  |
|  |  | (0.004) |  |  |
| Constant | -0.040 | -0.046 | -0.058 | -0.041 |
|  | (0.011)*** | (0.012)*** | (0.029)** | (0.012)*** |
|  |  |  |  |  |
| *R*^2^ | 0.11 | 0.11 | 0.17 | 0.10 |
| *N* | 2,349 | 2,326 | 445 | 1,940 |

Note. The table reports OLS estimates with robust standard errors. Standard errors are reported in parenthesis. ***, **, and * indicate statistical significance at the 1%, 5% and 10% confidence level, respectively.

**B. The Income and Spending Expectations (ISEC) Survey**

The Income and Spending Expectations (ISEC) survey conducted during the Covid-19 pandemic, aimed to collect information on households’ expectations about individual and aggregate income, their employment status, and their health-related experience of Covid-19 to enable comparison with their expectations about consumption in 2022. The questionnaire also includes data related to socioeconomic variables such as age, household size and composition, education, and occupation status. ISEC covers a representative sample of the Italian resident population, aged between 18 and 75. The 3,016 individuals in the sample were interviewed online by Doxa, a leading Italian firm that is engaged in market research and social studies.

**Sample design.** The sample is drawn from a larger representative sample of 120,000 individuals, maintained and updated regularly by Doxa. The interviews were enabled by a Computer Assisted Web Interviewing (CAWI) method. The overall response rate was 71.2%, with low levels of non-response for all questions. The questionnaire was constructed with the help of field experts and academic researchers and was piloted with 100 respondents in the first two weeks of November 2021. The sample is stratified by gender, age (18-24, 25-34, 35-44, 45-54, 55-64, 64-75), and geographical area (North-West, North-East, Centre and South and Islands). The survey technique used was CAWI (Computer Assisted Web Interviewing). The survey took place between 20 November and 5 December 2021.

**Demographic variables.** We compare the demographic and occupational characteristics of the ISEC initial sample with the SHIW 2020. Table 1 presents the means of main demographic variables in the ISEC (column 1) and SHIW (column 3) samples. The share of men/women is close. The age distributions are similar for the first three classes, covering individuals aged 18 to 54 years old, but the group of 65+ is 19% in the SHIW and 12% in ISEC. There is a significant difference between these two surveys with respect to education level: in ISEC survey, the share of respondents with primary education only is 19% and the share with tertiary education is 26%, compared to respectively 40% and 18% in the SHIW. Household size is similar in both, although, the share of single households in SHIW is larger. In terms of geographical distribution, the shares of households living in the North, South and Centre of Italy are almost identical in the two samples.

**The Doxa Panel.** The Doxa panel is a web platform designed and developed by Doxa to respond specific research needs. Doxa’s proprietary panel has over 120,000 registered panelists, 50,000 of which are active (completed a survey in the previous 12 months or signed up in 2021). Average response rate was 40% and invitations to respond to the survey were sent to users, on average, 2.5 times a month (around 33% are via mobiles).

**Recruitment of panel.** Doxa carries out periodic subscriber recruitment (2-3 times a year) to widen the reference base for online searchers and guarantee rotation of subscribers (10%-20% of active panel users are replaced each year). Particular attention is paid to representativeness of the panel. Several strategies are implemented to reduce distortion in the panel recruitment process, including annual offline and online recruiting.

**Incentives for panel.** Subscribers to web panels receive incentives for active participation in the proposed research. Doxa pays close attention to the type of incentive system since it could affect the decision to join the panel and result in self-selection problems, behavior/attitudes when responding to questions and, thus, the result survey. To filter out participants interested only in the incentive, a donation to a non-profit charity is associated with payment of the personal incentive.

**Fieldwork management.** During the fieldwork phase, Doxa follows rigorous procedures to limit bias introduced by fast respondents or speeders. The questionnaires are administered randomly to participants; invitations are staggered across several days to try to reduce speeders; the invitation remains valid for at least a week (including a weekend) to allow participation of individuals who do not look at their email every day and helping to ensure participation of individuals who tend not to reply immediately.

**Variables used in the estimation.** We report the wording of the relevant variables used in the estimation. Each question allows “don’t know” and “prefer not to answer”:

*Macroeconomic forecast* (qualitative). In your opinion, in 2022, the general economic situation will be: much worse; slightly worse; more or less the same; slightly better; much better.

*Macroeconomic forecast* (quantitative). In your view, how much will the Italian economy grow next year (2022)? Responses are coded as: less than -2%; -2; 0; 1; 2; 3; 4; 5; 6; 7; 8; 9; 10; above 10%.

*Health forecast* (quantitative). In your view, what percentage of Italians (aged over 12) will have received a booster shoot (third dose) by Jan 2022? Responses are coded as: 0-5; 5-10; 10-15; 15-20; 20-25; 25-30; 35-40; 40-45; above 50.

*Health forecast* (qualitative). In your opinion, in 2022, the general health situation will be: much worse; slightly worse; more or less the same; slightly better; much better.

*Infection fear*. In the context of the current health crisis, can you indicate, on a scale from 1 to 10 where 1 means “not worried at all” and 10 mean “extremely worried”, how worried you are about catching or passing on Covid-19? Responses are coded on a 1 to 10 scale, separately for: while working; while shopping, eating out, or travelling; contact with household members; contact with relatives and friends; fear of infecting others.

*Probability of lockdown*. On a scale from 1 to 10 (unlikely to extremely likely), rank your expected probability of a further lockdown.

*Covid-19 experience*. Which of the following items best describes your and your household’s experience of Covid-19? (You may indicate more than one item): I was infected; a member of my household was infected; no one in the household was infected.

*Vaccination*. Which of the following best describes your experience of the vaccination program: I have received my first shot; I have received my second shot; I have received my booster shot; I have not been vaccinated.

*Expected income* *growth*. In 2022, you expect your household disposable income, compared to 2021, will

|  | Distribute 100 points in the following table |
| --- | --- |
| Decrease by more than 10% |  |
| Decrease by between 5% and 10% |  |
| Decrease by between 0% and 5% |  |
| Be approximately the same |  |
| Increase by between 0% and 5% |  |
| Increase by between 5% and 10% |  |
| Increase by more than 10% |  |
| Total | 100 |

*Expected consumption growth.* The format used for expected income was also used for expected household’s home food consumption, food consumption away from home, online purchases, total consumption.

*Employment probability* (if employed). Rank the likelihood that you will be in employment in your current job in the next 12 months, from 1 (extremely unlikely) to 10 (extremely likely).

*Employment probability* (if looking for your first job or unemployed). Rank the likelihood that you will find a job in the next 12 months, from 1 extremely unlikely to 10 extremely likely.
